# CFD^+^CD14^+^ monocytes: potential pathogenic subset in myasthenia gravis uncovered by multi-omics integration and machine learning analysis

**DOI:** 10.3389/fimmu.2026.1813107

**Published:** 2026-05-01

**Authors:** Shuang Li, Yifan Zhang, Chenlu Hou, Ting Chang

**Affiliations:** 1 Department of Neurology, Tangdu Hospital, the Fourth Military Medical University, Xi’an, Shaanxi, China; 2 School of Medicine, Northwest University, Xi’an, China

**Keywords:** CFD, machine learning, myasthenia gravis, scRNA-seq, TWAS

## Abstract

**Background:**

Transcriptome−wide association study (TWAS) contributes to discovering novel susceptibility genes related to diseases. Single-cell RNA sequencing (scRNA-seq) has been extensively applied to characterize cellular heterogeneity in various biological and pathological conditions. However, few studies have integrated TWAS and scRNA-seq to decode the pathogenesis of myasthenia gravis (MG) at cellular resolution.

**Methods:**

We integrated multi-omics data from GEO, genome-wide association study (GWAS) catalog, and Genotype-Tissue Expression (GTEx) databases, including scRNA-seq data, GWAS summary data, and expression quantitative trait loci. A set of risk genes genetically associated with MG were identifies using TWAS analysis. TWAS activity status across cell types in MG at single cell resolution was assessed based on these genes. Seven machine learning algorithms were performed to identify high TWAS activity related signature genes. Machine learning benchmark was incorporated to select the best model using R package “mlr3”. Meanwhile, we performed scRNA-seq on peripheral blood collected from MG patients and healthy controls to generate a validation dataset.

**Results:**

scRNA-seq analysis revealed an elevated proportion of CD14^+^ monocytes in MG patients, accompanied by notable transcriptomic reprogramming. Compared with other cell types, CD14^+^ monocytes exhibited higher TWAS activity. High TWAS activity subsets mainly enriched in the MG group and functionally associated with mTORC1 signaling, complement, and inflammatory response pathways. Machine learning-based feature selection revealed six robust signature genes (FKBP15, EHMT1, CHPT1, KLC1, SCPEP1, and CFD). Among these, CFD showed elevated expression early in high TWAS activity monocyte development. Compared to control group, CFD maintained higher levels throughout disease progression in MG group. CFD^+^CD14^+^ monocytes exhibited enhancer plasticity than CFD^-^ CD14^+^ monocytes and were related to complement and coagulation cascades, antigen processing and presentation, and proteasome pathways. CFD^+^CD14^+^ monocytes in MG exhibit enhanced intercellular communication activity compared to CFD^-^ cells, demonstrating greater signal reception and transmission capacity.

**Conclusion:**

In summary, this study systematically delineates the important role of CFD^+^CD14^+^ monocytes in MG pathogenesis through multi-omics analysis, providing novel insights for a deeper understanding of MG immunopathology and potential therapeutic targets.

## Introduction

Myasthenia gravis (MG) is a prototypical autoimmune disorder characterized by autoantibody-mediated impairment of neuromuscular transmission, leading to fatigable muscle weakness (1). While the pathogenic role of B cells and autoantibodies against the acetylcholine receptor (AChR) is well-established (2), the precise cellular mechanisms initiating and perpetuating the aberrant immune response remain incompletely understood, particularly the complex crosstalk between innate and adaptive immune compartments. Therefore, a deeper resolution of the immune dysregulation, particularly within innate immune compartments, is crucial for developing targeted therapies.

Innate immune cells, particularly monocytes, have emerged as central regulators of autoimmune pathogenesis (3). Among monocyte subsets, CD14^+^ monocytes are critical for initiating and amplifying inflammatory responses through cytokine production, antigen presentation, and activation of adaptive immune cells⁠ (4). Recent single-cell RNA sequencing (scRNA-seq) studies have reported altered proportions and functional dysregulation of CD14^+^ monocytes in MG patients (5–7), but the molecular cues driving their pathogenic reprogramming remain unclear. Additionally, genome-wide association studies (GWAS) have identified multiple MG susceptibility loci (8–10), but translating these genetic associations into cell-type-specific functional mechanisms has been challenging due to the lack of integrative multi-omics approaches.

Transcriptome-wide association studies (TWAS) have emerged as a powerful approach to bridge the gap between genetic associations and functional biology by identifying genes whose genetically regulated expression is associated with disease risk (11, 12). This method has successfully nominated novel candidate genes in various autoimmune diseases. Concurrently, scRNA-seq has revolutionized our ability to deconvolute cellular heterogeneity and identify novel cell states within complex tissues, providing unparalleled insights into disease-specific alterations at a cellular level (13, 14). For instance, scRNA-seq studies in multiple sclerosis and rheumatoid arthritis have revealed pathogenic immune cell subsets undetectable by bulk analyses (15, 16). Despite these advances, the integration of TWAS findings with single-cell resolution transcriptomics to elucidate cell type-specific mechanisms in MG is notably lacking. This limits our understanding of how genetically influenced gene expression programs operate within specific immune cell populations to drive MG pathology.

In this study, we integrated multi-omics data from public repositories (GEO, GWAS catalog, GTEx) with an independent in-house scRNA-seq cohort of MG patient blood. TWAS analysis was performed to systematically identify genetically risk genes associated with MG. Combining with scRNA-seq data, we identified a unique CD14^+^ monocyte subtype with high TWAS activity. Multi-faceted analyses confirmed high TWAS activity CD14^+^ monocytes exhibited functional reprogramming, which might exert potentially important role in the development of MG. Through integrating machine learning and in-house scRNA-seq data analyses, we found that CFD might mediate the functional regulation of CD14^+^ monocytes in the pathogenesis of MG. Our integrative approach bridges genetic associations and cellular heterogeneity, providing novel insights into MG pathogenesis at a cellular level and identifying new therapeutic targets.

## Methods

### Data acquisition

The raw data of single-cell RNA-seq profile under accession number GSE227835 (17), which included 10 MG samples with anti-AChR antibody positive (anti-AChR^+^) and 10 healthy controls, was downloaded from GEO database (http://www.ncbi.nlm.nih.gov/geo). GWAS summary data (GCST90093061) of MG including 1873 anti-AChR^+^ MG patients and 36370 healthy controls was downloaded from GWAS catalog (https://www.ebi.ac.uk/gwas/). Gene expression weights of whole blood from Genotype-Tissue Expression (GTEx) version 8 were used for transcriptomic imputation and association testing.

### Sample collection and processing

Peripheral blood samples from treatment-naïve MG patients and healthy controls were collected and processed for single cell RNA sequencing (scRNA-seq) using Singleron platform. Red blood cells of peripheral blood were removed with red blood cell lysis buffer. Proceed single-cell suspension were then loaded into the microwell chip, preparing 5’ gene expression library. The resulting scRNA-seq libraries were sequenced on an Illumina Novaseq 6000 platform with 150 bp paired end reads. Raw reads from scRNA-seq were processed to generate gene expression matrixes using CeleScope pipeline (https://github.com/singleron-RD/CeleScope/releases). The clinical information for MG patients was showed in **Supplementary Table 1**.

For RT-PCR, a total of 13 MG patients were enrolled for collecting peripheral blood from the Department of Neurology at Tang Du Hospital. Meanwhile, 13 age- and sex-matched healthy controls were selected among volunteers. Peripheral blood samples collected from of subjects were put in tubes containing ethylenediaminetetraacetic acid. Peripheral blood mononuclear cells (PBMCs) were further isolated using lymphocyte separation. Both MG patients and healthy controls signed a consent form for the collection of data and blood samples. The study was approved by the local ethics committee.

### Single cell RNA-seq data processing

GSE227835 dataset and scRNA-seq data from our team were analyzed using Seurat (V5) R package. For the initial quality control (QC) step, cells with expression of < 200 or > 6000 genes and more than 15% mitochondrial counts were filtered out of the analysis. The top 2000 highly variable genes were identified by FindVariableFeatures function. Subsequently, we performed standard data scaling using the ScaleData function followed by principal component analysis (PCA) using RunPCA, projecting the highly variable genes into a 50-dimensional PCA space. The Harmony R package (group.by.vars = “sample”) was used to reduce both batch effects and biases between different patients. The Uniform Manifold Approximation and Projection (UMAP) embedding and neighborhood graph were calculated using the first 20 dimensions generated by Harmony. Clustering was performed with optimal resolution. For the subsequent analysis (including differential analyses, pathway analyses, and TWAS activity comparisons), the cell is treated as the statistical unit to account for patient-level variation.

### Transcriptome−wide association study

We performed transcriptome-wide association study (TWAS) using the FUSION pipeline. This method integrates GWAS summary statistics with gene expression reference data to predict tissue-specific gene expression and identify phenotype-associated expression changes (12). Precomputed gene expression weights on GTEx (version 8) data from whole blood were used to estimate a gene’s association to MG risk. To account for multiple testing, we applied the Benjamini-Hochberg (BH) procedure to control the False Discovery Rate (FDR). The raw P-values (TWAS.P) were adjusted across all tested genes (n = 6245 genes) in the tissue. A threshold of FDR < 0.05 was used to define statistically significant gene-trait associations.

### Functional enrichment analysis

Gene Ontology (GO) and Kyoto Encyclopedia of Genes and Genomes (KEGG) functional enrichment analyses on TWAS genes and differentially expressed genes were performed using “clusterProfiler” R package (18). GO terms and KEGG pathways with p < 0.05 were considered as statistically significant. We performed Gene Set Variation Analysis (GSVA) using the “GSVA” R package (19) to quantify the enrichment of specific gene sets in each sample. This method calculates enrichment scores that reflect pathway activity, enabling assessment of signaling pathway enrichment across samples. The analysis utilized predefined gene sets from the “c5.all.v7.0.symbols.gmt” collection. Adjusted p-value < 0.05 was significant.

### Assessment of TWAS activity

Based on TWAS results (FDR < 0.05), 125 genes were identified (**Supplementary Table 2**) and defined as MG risk genes. In the downstream analysis, these genes as the input set were used to calculate a “TWAS activity score” per cell, which reflects the combined expression of MG risk genes. AddModuleScore, AUCell, UCell, singscore, and ssGSEA algorithms were further performed to evaluate and determine the TWAS activity of each cell at single-cell level. The raw score matrix of TWAS activity was processed in two sequential steps. First, Z-score standardization was applied to transform each feature to a distribution with zero mean and unit variance. Subsequently, a custom Min-Max normalization function was employed to linearly rescale the values of each feature column to a [0, 1] range, ensuring consistency across all features. The processed matrix was converted to a data frame. A composite TWAS score (Scoring) was then calculated for each row by summing the normalized feature values across all columns, that is the sum across the five normalized scores. Cells were stratified into three groups based on composite score (Scoring) quartiles: low TWAS activity state (≤25th percentile), median activity state (25th–75th percentile), and high TWAS activity state (≥75th percentile). High TWAS activity indicates that there is a higher composite expression level of MG risk genes in a given cell, making them candidate cellular drivers of disease.

### Identification of high TWAS activity signature genes

To identify high TWAS activity related feature genes and further assess the contribution of different TWAS genes to the Scoring, we integrated a panel of machine learning algorithms, including the Least Absolute Shrinkage and Selection Operator (LASSO), Random Forest (RF), eXtreme Gradient Boosting (XGBoost), Gradient Boosting Machine (GBM), Boruta algorithm, Decision Tree (DT), and Adaptive Barycentric Coordinate Regression and Classification (ABESS). The LASSO regression (20) conducts variable selection and shrinks coefficients through regularization, thereby mitigating overfitting and improving the generalizability of predictive models. RF (21), a powerful and widely adopted strategy for diverse prediction problems, was utilized to identify key genes in this study. XGBoost (22) is a highly optimized gradient-boosting framework that excels at processing large-scale, complex datasets while also mitigating overfitting and enhancing generalization. GBM (23) leverages ensembles of weak learners to iteratively improve prediction accuracy. The Boruta algorithm (24) is employed to elucidate the key factors influencing the dependent variable. DT (25) is a supervised classifier that uses a tree-like structure, where internal nodes test attributes, branches denote outcomes, and leaf nodes assign class labels to solve categorization problems. ABESS (26) is an efficient algorithm for large-scale linear regression and classification that performs adaptive feature selection to enhance model performance. Finally, we employed receiver operating characteristic (ROC) curves to further validate the discriminatory ability of the candidate genes.

### Machine learning benchmark for predicting high TWAS activity signature

To establish an accurate and robust high TWAS activity signature in scRNA-seq level, R package “mlr3” was incorporated to select the best model. In this analysis, we first excluded cells with median TWAS activity and retained only those with high or low TWAS activity as the prediction target and control groups, respectively. The feature matrix consisted of cells × 125 TWAS risk genes, where the expression values were normalized log−transformed data (slot = “data”) extracted from the Seurat object. The outcome label was a binary variable: cells with TWAS_activity == “high” were labeled as 1 (case), and cells with TWAS_activity == “low” were labeled as 0 (control). The final dataset contained approximately balanced numbers of high and low TWAS activity cells (ratio ~1:1). The dataset was randomly split into a training set (80%) and an independent test set (20%) using stratified sampling based on the outcome label to preserve class proportions. Importantly, to avoid data leakage, we ensured that all cells from a given patient were assigned entirely to either the training set or the test set, not split across both. This was achieved by first grouping cells by patient identifier (orig.ident) and then assigning entire patients to the training or test set. We employed nested cross-validation, with inner 5-fold CV for hyperparameter tuning and outer 10-fold CV for model evaluation. The model with the highest mean AUC was deemed optimal. To further assess whether the candidate gene signature generalizes across independent individuals rather than learning patient-specific patterns, we performed leave-one-patient-out cross-validation (LOPO-CV). In this scheme, each patient was held out as the test set exactly once, while cells from all other patients were used as the training set. For each iteration, we trained a logistic regression model using the candidate signature genes as predictors and TWAS activity status as the binary outcome. The probability of high TWAS activity was predicted for cells in the held-out patient, and the AUC was calculated. Patients whose test set contained only one class (either all high or all low TWAS activity cells) were excluded from the analysis. This process was repeated for all patients.

### Cellular interaction analysis

Cellular interaction analysis was conducted by CellChat v2.1.2 following the official tutorial. The significant cell-cell interactions were identified with default parameters and each interaction was assigned to a probability value. “netAnalysis_signalingRole_heatmap” function was utilized to visualize the differences of incoming and outgoing signaling pathways between control and MG groups. “netAnalysis_signalingRole_scatter” was used to identify cell populations with significant changes in incoming and outgoing interaction strength. The “netVisual_bubble” function was employed to display the increased signaling pathways between CFD^+^ monocytes and B cells in MG group.

### Pseudotime analysis

Two algorithms including Monocle2 (27), CytoTRACE 2 (28), were used to infer the pseudotime trajectory of cell types. Monocle2 employs a robust dimensionality reduction algorithm that effectively maps high-dimensional single-cell RNA sequencing data to a low-dimensional space. This enables the visualization of dynamic changes in cell states and the inference of potential differentiation trajectories. CytoTRACE 2 could predict cell fate without relying on prior knowledge, which exploited the well-established principle that transcriptional diversity declines during differentiation to predict cellular differentiation states from scRNA-seq data.

### RT-qPCR analysis

Total RNA extractions were performed using the Total RNA Kit (Omega, USA), following the provided protocol. RNA concentration and quality were assessed using NanoDrop 2000 (Thermo Fisher Scientific, Illkirch-Graffenstaden, France). Synthesis was carried out using the FastKing gDNA Dispelling RT SuperMix (Tiangen Biotech, China), which also served as the template for RT-qPCR. The amplifications were performed using the SuperReal PreMix Plus (SYBR Green) on a CFX96 Real-Time PCR detection system (all from Bio-Rad Laboratories, Hercules, CA). The 2^−ΔΔCt^ method was used for the calculation of relative expression levels normalized by GAPDH.

## Results

### TWAS analysis identified MG related risk genes

**Figure 1:**
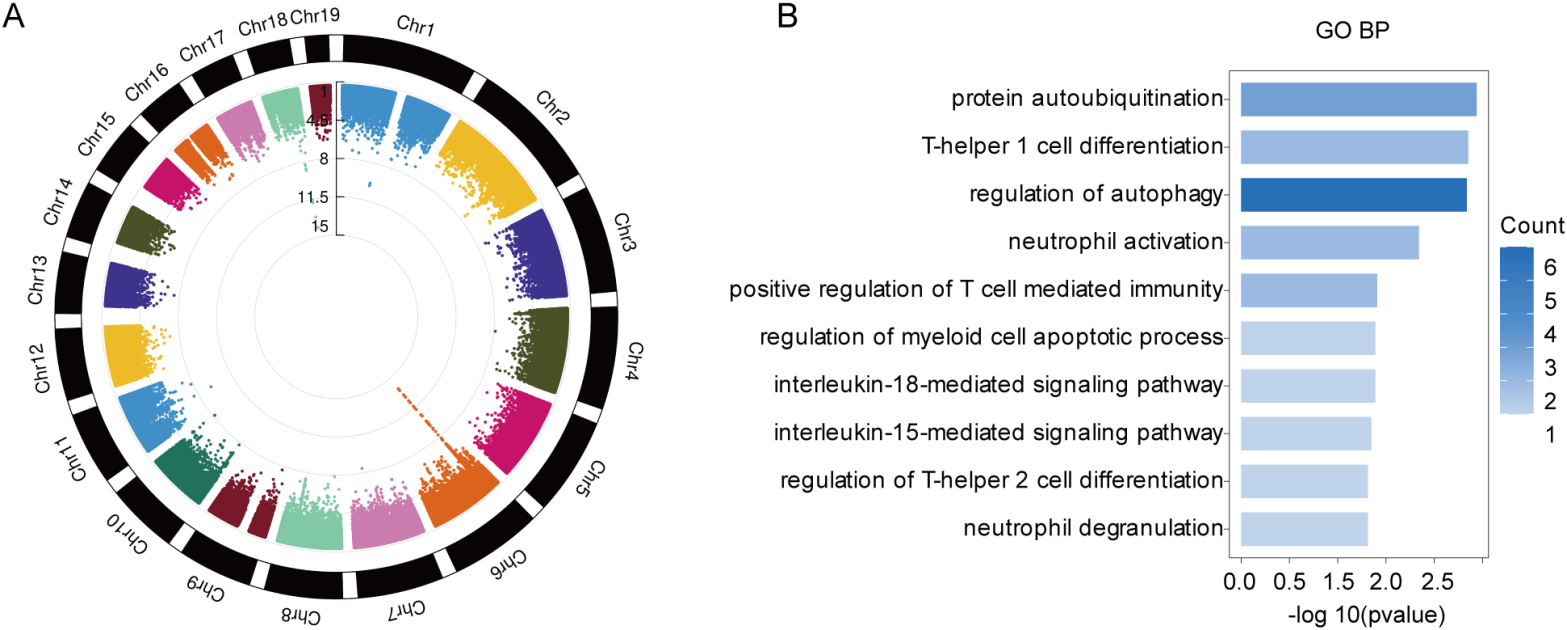
Identification of risk genes associated with MG using TWAS analysis. **(A)** Circular Manhattan plot of TWAS risk genes for MG. **(B)** GO terms (p < 0.05) enriched by TWAS risk genes.

According to the TWAS analysis, we obtained 125 genes associated with MG (**Figure 1A**; **Supplementary Figure 1**). To further explore the biological function of these TWAS genes, we performed GO enrichment analysis. These risk genes were enriched in immune response related GO terms, such as T helper 1 cell differentiation, T helper 2 cell differentiation, T cell mediated immunity, and neutrophil activation/degranulation (**Figure 1B**), suggesting TWAS genes may play important roles in the pathogenesis of MG.

### scRNA-seq analysis revealed notably transcriptomic change of CD14^+^ monocytes in MG

**Figure 2:**
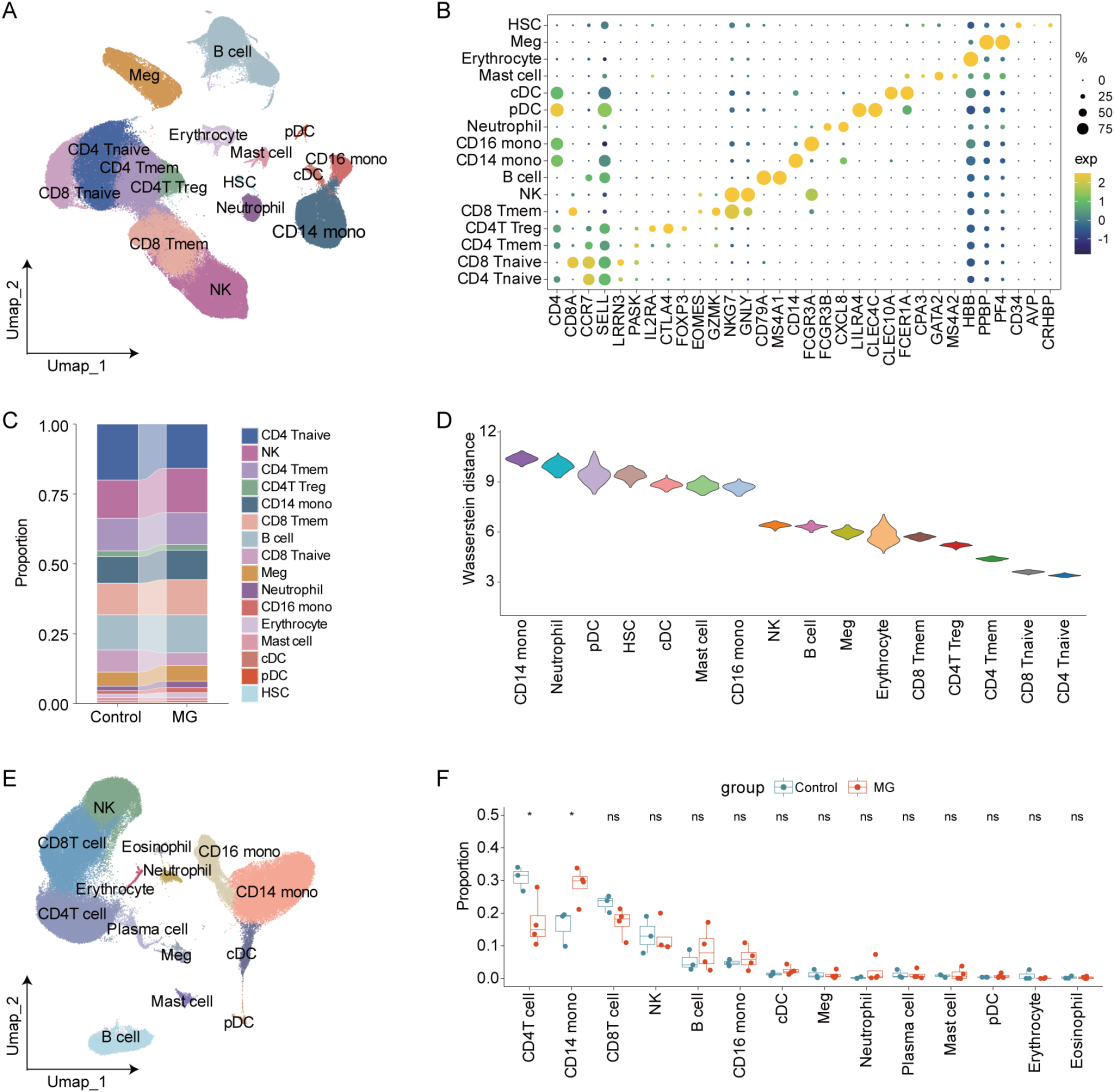
Notably transcriptomic change of CD14^+^ monocytes in MG. **(A)** UMAP showing major cell types in GSE227835 dataset. **(B)** Dot plot showing known gene markers. **(C)** The proportions of major cell types in patients with MG vs controls. **(D)** Optimal transport analysis based on Wasserstein distance for each cell type. **(E)** UMAP showing major cell types in in-house scRNA-seq data. **(F)** The proportion difference of each cell type between MG vs control (used in-house scRNA-seq data). *p < 0.05; ns, not significant.

Following quality control, a total of 201,941 high-quality cells from 10 MG patients and 10 healthy controls in the GSE227835 dataset were retained for subsequent analysis. The cells were further annotated into 16 cell types, including CD4^+^ naive T (CD4 Tnaive), CD4^+^ memory T cells (CD4 Tmem), CD4^+^ Treg cells (CD4 Treg), CD8^+^ naive T (CD8 Tnaive), CD8^+^ memory T cells (CD8 Tmem), B cells, NK cells, CD14^+^ monocytes (CD14 mono), CD16^+^ monocytes (CD16 mono), neutrophils, plasmacytoid dendritic cells (pDC), conventional dendritic cells (cDC), mast cells, hematopoietic stem cells (HSC), erythrocytes, megakaryocytes (**Figure 2A**; **Supplementary Figures 2A, B**). Cell types were identified based on known canonical marker genes (**Figure 2B**). Although the statistical differences were not significant, CD14^+^ monocytes displayed an increasing trend in MG samples compared with controls (**Figure 2C**; **Supplementary Figure 3**). Optimal transport analysis revealed that CD14^+^ monocytes exhibited the largest Wasserstein distance (**Figure 2D**), indicating their profound transcriptomic reprogramming in MG. Additionally, we performed single-cell transcriptomic profiling using peripheral blood samples from 4 MG patients and 3 healthy controls. We identified the same major cell types as those in the GSE227835 dataset, such as T cells, B cells, NK cells, CD14^+^ monocytes, CD16^+^ monocytes and neutrophils (**Figure 2E**; **Supplementary Figures 2C, D**). Notably, the proportion of CD14^+^ monocytes was also elevated in our scRNA-seq data (**Figure 2F**). These results collectively indicated that CD14^+^ monocytes played critical roles in the pathogenesis of MG.

### Characterizing the complexity and heterogeneity of TWAS activity

**Figure 3:**
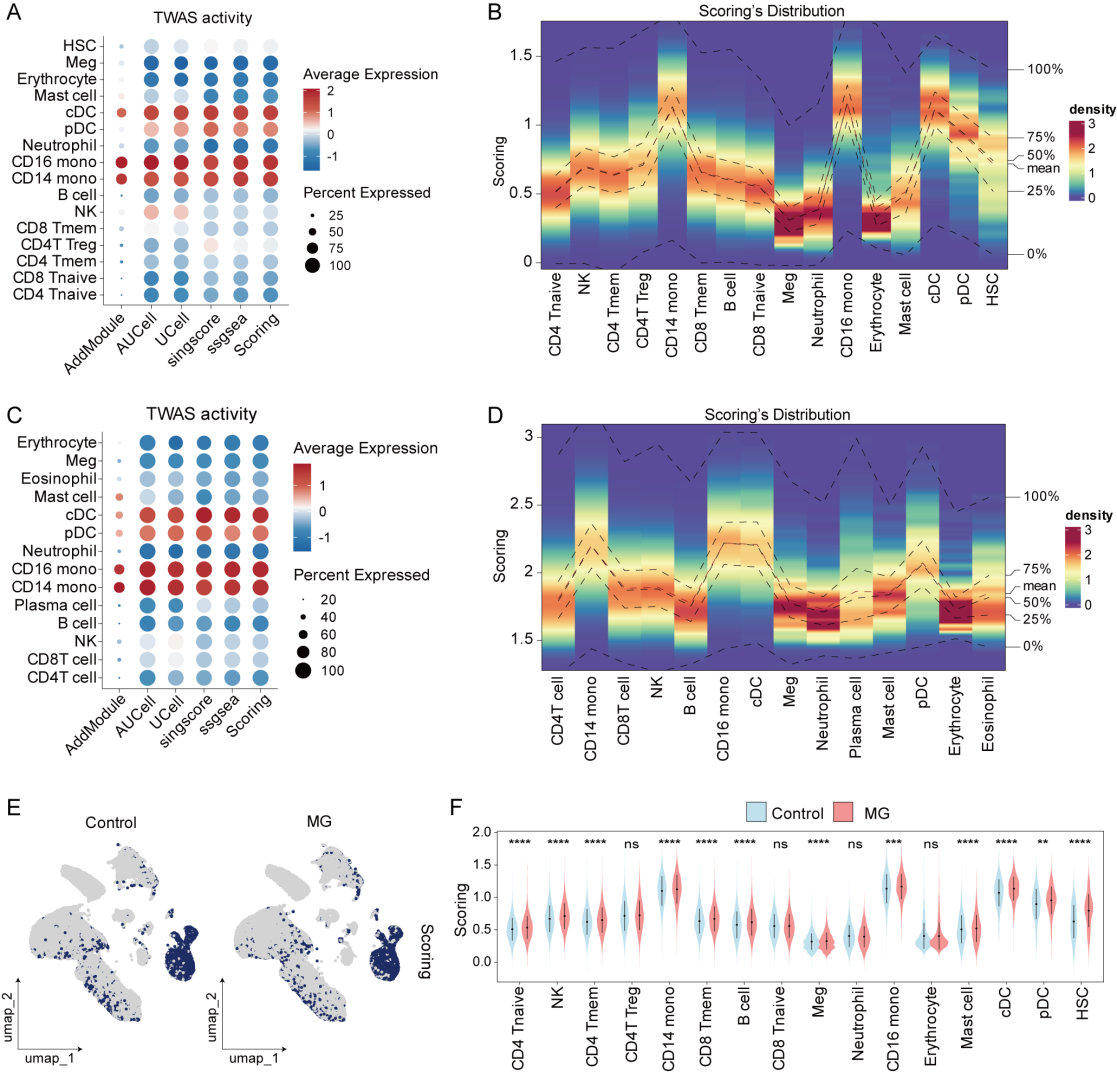
The distribution of TWAS activity between MG vs control group. **(A)** Dot plot showing the TWAS activity scores in GSE227835 dataset. **(B)** Heatmap showing the distribution of scoring for major cell types in GSE227835 dataset. **(C)** Dot plot showing the TWAS activity scores in in-house scRNA-seq data. **(D)** Heatmap showing the distribution of scoring for major cell types in in-house scRNA-seq data. **(E)** Umap plot showing the distribution of TWAS activity scoring in both MG and control samples. **(F)** The difference of TWAS activity scoring for each cell type between MG vs control. **p < 0.01, ***p < 0.001, ****p < 0.0001; ns, not significant.

To delineate the complexity and heterogeneity of TWAS activity in MG, we employed five algorithms, including AddModuleScore, AUCell, UCell, singscore, and ssGSEA, to quality the activity scores for each cell. We revealed distinct TWAS activity patterns across cellular strata, finding CD14^+^ monocytes to be strikingly elevated compared to other cell types (**Figures 3A, B**). The correlation analysis indicated that the TWAS activity scores of the 5 algorithms were positive correlated (**Supplementary Figure 4**). This pattern was validated by our independent scRNA-seq data (**Figures 3C, D**). Furthermore, comparison of MG versus control groups revealed a broader state distribution of TWAS activity in MG, alongside substantial variations across its cellular subtypes (**Figures 3E, F**).

### High TWAS activity CD14^+^ monocytes exhibited elevated plasticity

**Figure 4:**
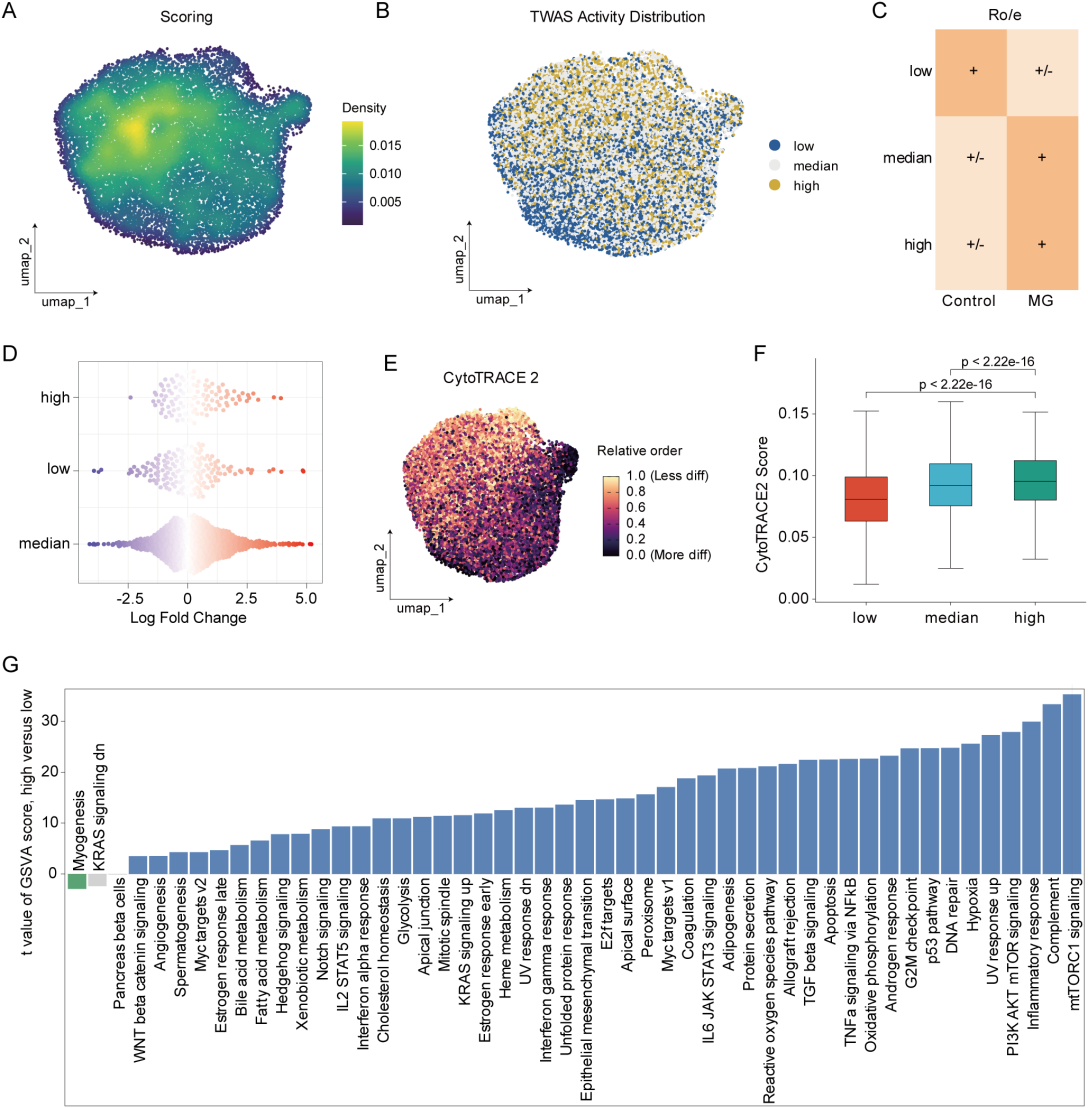
Identification of high TWAS activity subpopulation. **(A)** The density distribution of TWAS activity in CD14^+^ monocytes. **(B)** UMAP plot showing three TWAS activity status of CD14^+^ monocytes (including high TWAS activity, median TWAS activity, and low TWAS activity). **(C)** Intergroup enrichment comparison revealed by Ro/e (ratio of observed cell number to expected cell number) for each TWAS activity status. **(D)** miloR analysis displaying the enrichment of TWAS activity status between MG vs control group. **(E)** CytoTRACE analysis of TWAS activity status. **(F)** Comparison of CytoTRACE score among three TWAS activity status. **(G)** The enrichment of hallmark genesets in high and low TWAS activity using GSVA (p.adjust < 0.01).

The observed elevation of TWAS activity in CD14^+^ monocytes prompted further investigation. This analysis demonstrated a heterogeneous density distribution, with diverse activity levels across the cellular population (**Figure 4A**; **Supplementary Figure 5**). We further observed that variations in TWAS activity among CD14^+^ monocyte subpopulations corresponded to discrete spatial localization in the UMAP embedding (**Figure 4B**). Ro/e analysis was performed to quantify the enrichment of distinct activity CD14^+^ monocytes. Among all cells, high TWAS activity CD14^+^ monocytes were predominantly found in MG group (**Figure 4C**). MiloR analysis further confirmed that high TWAS activity monocytes enriched in MG group (**Figure 4D**). cytoTRACE analysis was subsequently applied to investigate the basis of heterogeneous TWAS activity among CD14^+^ monocytes. Although monocytes in peripheral blood are all in a mature state, cytoTRACE analysis revealed that those with high TWAS activity also exhibited elevated cytoTRACE scores and showed spatial concordance in the UMAP projection (**Figures 4E, F**). To delineate the biological mechanisms underlying differential TWAS activity, we performed GSVA. The analysis identified pronounced enrichment of mTORC1 signaling, complement, and inflammation response pathways in the high TWAS activity group (**Figure 4G**). These results implied that monocytes in MG undergo reprogramming, acquiring a more plastic functional state.

### Identification of high TWAS feature genes with multiple machine learning algorithms

**Figure 5:**
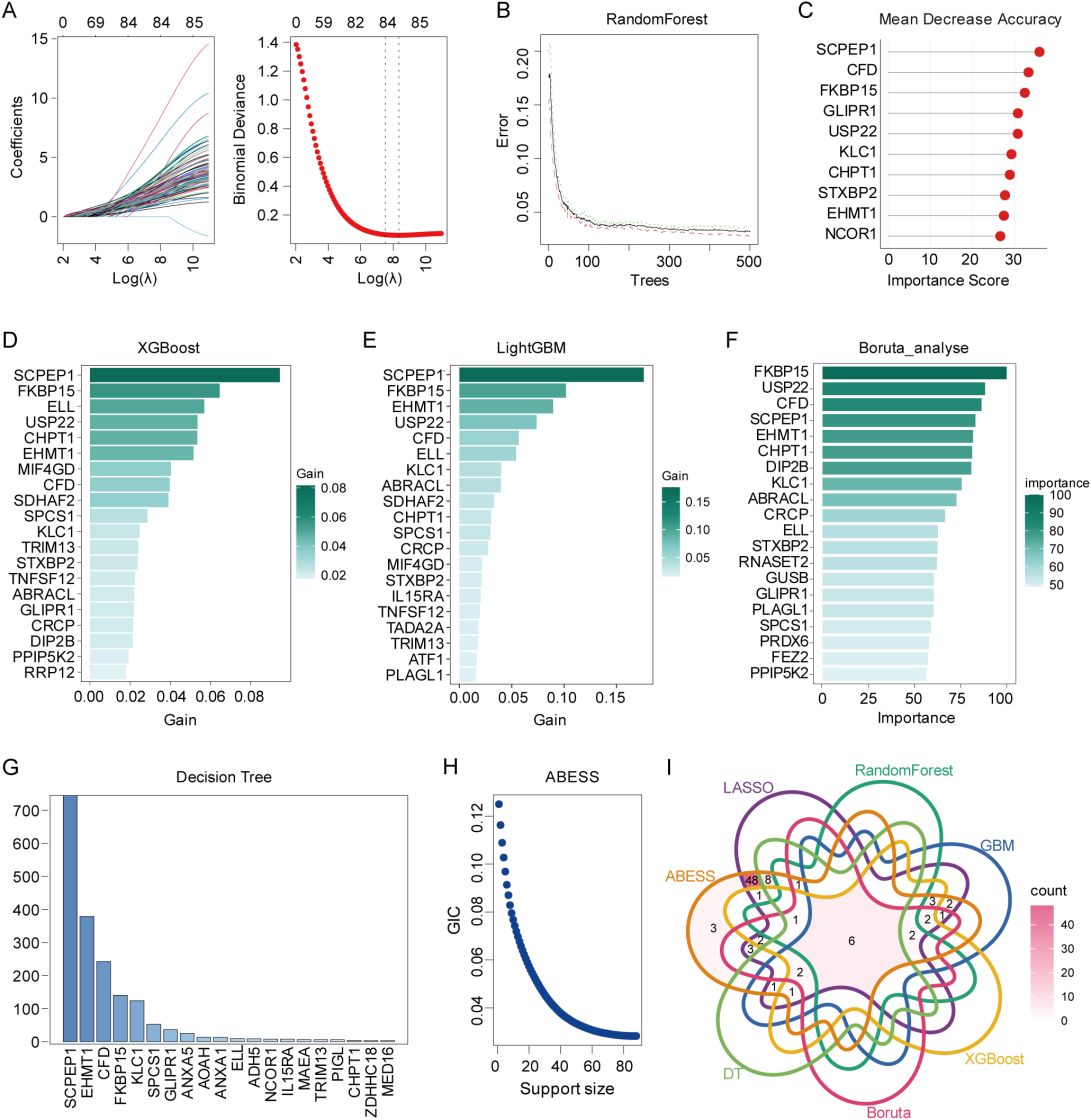
Multiple machine learning algorithms identified feature genes associated with TWAS activity based on GSE227835 dataset. **(A)** LASSO coefficient profiles for the selected feature genes (λ selected at minimum deviance). **(B)** RF for the relationships between the number of trees and error rate. **(C)** Importance score of feature genes calculated by RF. **(D)** The importance of feature genes identified using XGBoost algorithm. **(E)** The importance of feature genes calculated using GBM algorithm. **(F)** The importance of feature genes calculated using Boruta algorithm. **(G)** DT algorithm displayed the importance of feature genes. **(H)** The further select candidate optimal feature genes through ABESS algorithm. **(I)** Venn diagram showing the six feature genes shared by the above algorithms.

To further identify robust high TWAS feature genes from the 125 risk genes, we employed a binary classification framework. Cells exhibiting high TWAS activity were designated as the prediction target, while low TWAS activity cells served as the control group. Using LASSO regression, we identified 84 feature genes with non-zero coefficients (coefficient > 0) associated with high TWAS activity (**Figure 5A**; **Supplementary Table 3**). Based on the Random Forest (RF) algorithm, we identified the top 10 feature genes with importance score > 26 (**Figures 5B, C**; **Supplementary Table 4**). Through application of the GBM (**Figure 5D**; **Supplementary Table 5**) and XGBoost (**Figure 5E**; **Supplementary Table 6**) algorithms separately, we identified top 20 feature genes for each. Furthermore, after applying the Boruta algorithm to filter out irrelevant features, the top 20 features were selected as key genes. (**Figure 5F**; **Supplementary Table 7**). 20 feature genes were identified using the Decision Tree (DT) algorithm (**Figure 5G**; **Supplementary Table 8**). For ABESS algorithms, all genes identified by the model were retained as candidate feature genes (**Figure 5H**; **Supplementary Table 9**). Ultimately, six optimal feature genes (FKBP15, EHMT1, CHPT1, KLC1, SCPEP1, CFD) emerged from the intersection of results across all seven machine learning algorithms (**Figure 5I**).

### Expression and predictive accuracy of feature genes

**Figure 6:**
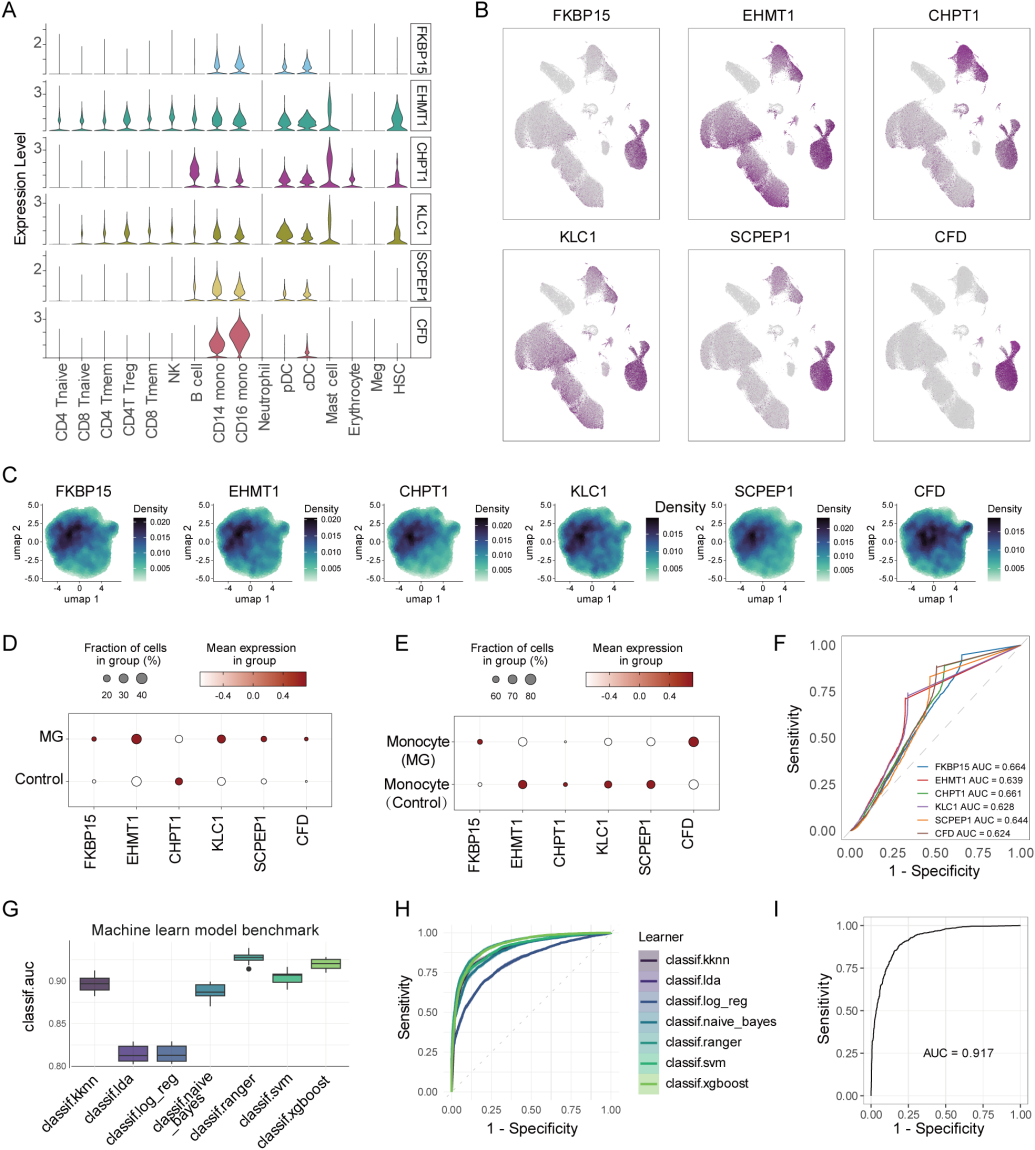
Evaluation and validation of the feature genes used GSE227835 dataset. **(A)** Violin plot showing the expression of 6 feature genes (FKBP15, EHMT1, CHPT1, KLC1, SCPEP1, CFD) at the scRNA-seq level. **(B)** UMAP plot showing the distribution of 6 feature genes in all cell types. **(C)** UMAP plot showing the density of 6 feature genes in CD14^+^ monocyets. **(D)** Dot plot showing the expression of feature genes between MG vs controls. **(E)** Dot plot showing the expression of feature genes in monocytes between MG vs controls. **(F)** ROC curves showing the diagnostic performance of 6 feature genes. **(G)** Mean AUC for machine learning model benchmark. **(H)** ROC curves showing machine learning model benchmark. **(I)** The ROC curve evaluated the accuracy of the model in test dataset.

To further evaluate the expression levels of the six candidate feature genes, we validate their expression patterns using scRNA-seq data. Compared to other cell types, all six feature genes were upregulated in myeloid cells, especially CD14^+^ and CD16^+^ monocytes (**Figures 6A, B**). Within the CD14^+^ monocyte population, these genes demonstrated pronounced spatial enrichment specifically in the high TWAS activity group (**Figure 6C**). Furthermore, we found that FKBP15, EHMT1, KLC1, SCPEP1, CFD were increased in MG than control group (**Figure 6D**). Among them, FKBP15 and CFD were upregulated in CD14^+^ monocytes of MG group (**Figure 6E**). To quantitatively assess the diagnostic and predictive value of the optimal feature genes, ROC curve analysis was performed and displayed in **Figure 6F**. To evaluate and compare the performance of different machine learning algorithms, we conducted a benchmarking analysis. The benchmarked models demonstrated excellent performance, achieving a mean AUC above 0.8 (**Figure 6G**). ROC curves for each individual model are presented in **Figure 6H**. Additionally, Precision-Recall curves were generated to further assess model performance across relevant metrics (**Supplementary Figure 6**). Finally, an overall ROC analysis of the benchmarking process itself confirmed high predictive accuracy, with an AUC of 0.917 (**Figure 6I**), which suggested six-gene signature significantly outperforms a single-gene model (**Figure 6F**). Finally, the LOPO−CV confirmed that the six−gene signature generalized well across independent patients and the per−patient AUC values were provided in **Supplementary Table 10**.

### Important role for CFD^+^CD14^+^ monocytes in MG development

**Figure 7:**
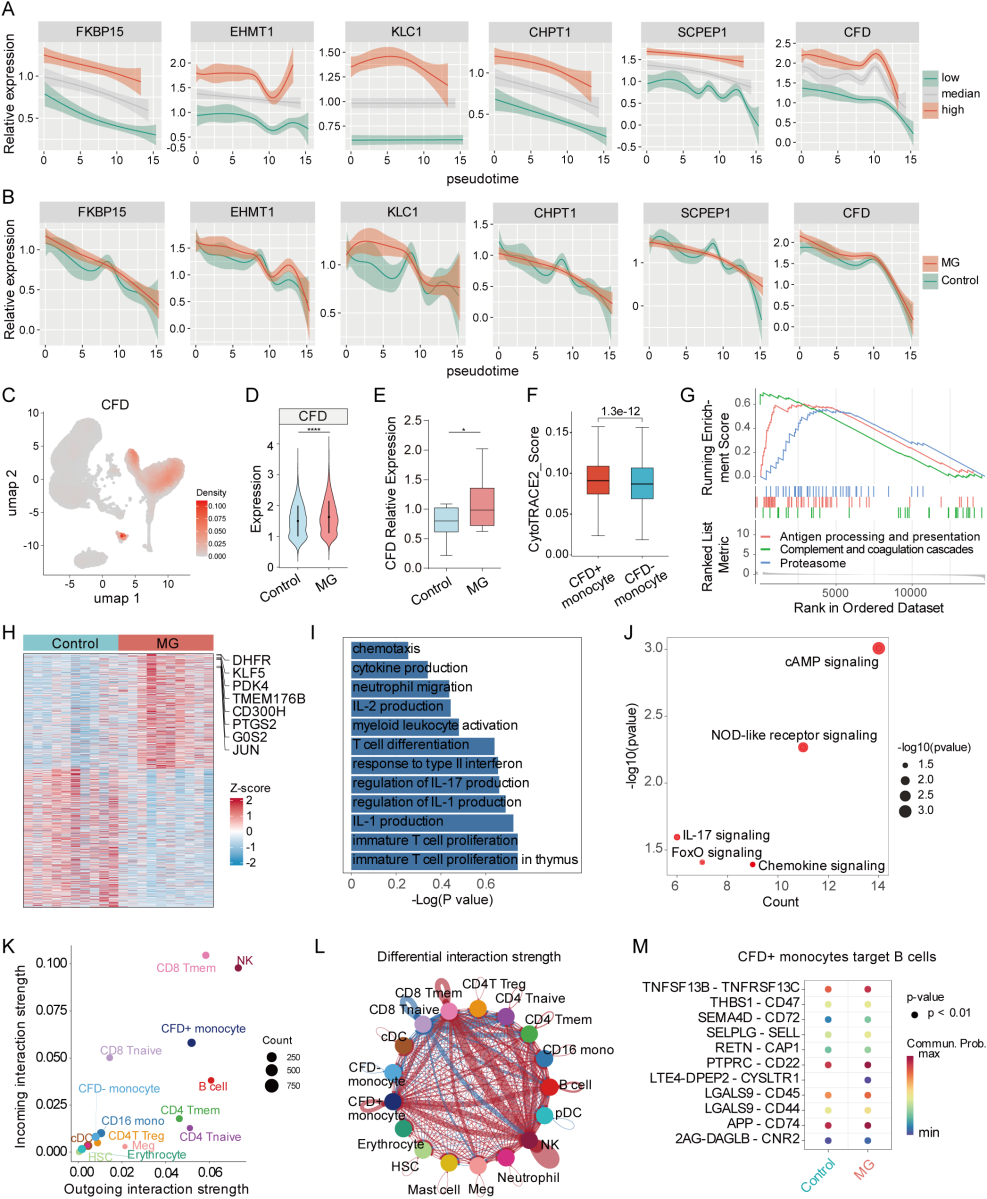
Functional analysis of CFD^+^CD14^+^ monocytes. **(A)** The expression of 6 feature genes among three TWAS activity in pseudotime analysis in GSE227835 dataset. **(B)** The expression of 6 feature genes between MG vs control group in pseudotime analysis in GSE227835 dataset. **(C)** UMAP plot showing the density distribution of CFD in in-house scRNA-seq data. **(D)** Violin plot showing the expression difference of CFD in CD14^+^ monocytes between MG vs control group. **(E)** The relative expression of CFD detected by RT-qPCR**. (F)** Comparison of CytoTRACE score between CFD^+^CD14^+^ monocytes and CFD^-^CD14^+^ monocytes in GSE227835 dataset. **(G)** Functional annotation of CFD^+^CD14^+^ monocytes using GSEA in GSE227835 dataset (p.adjust < 0.01). **(H)** Differentially expressed analysis of CFD^+^CD14^+^ monocytes between MG vs control group in GSE227835 dataset (p.adjust < 0.01). **(I)** GO terms enriched by upregulated genes of CFD^+^CD14^+^ monocytes (p < 0.05). **(J)** KEGG enriched by upregulated genes of CFD^+^CD14^+^ monocytes in GSE227835 dataset (p < 0.05). **(K)** The scatter plot of the inferred roles of cell types considering their incoming and outgoing interaction strength in GSE227835 dataset. **(L)** Differential interaction strength of all cell types between MG and groups in GSE227835 dataset. **(M)** Increased signaling between CFD^+^CD14^+^ monocytes and B cells in patients with MG in GSE227835 dataset. * indicates p < 0.05.

Pseudotime analysis was employed to assess the expression dynamics of TWAS feature genes (FKBP15, EHMT1, CHPT1, KLC1, SCPEP1, CFD) during monocyte development. The results revealed that CFD exhibited higher expression level than other genes, suggesting that CFD may be overactivated during early stage of MG (**Figure 7A**). Although CFD expression decreased with disease progression, its levels in the MG group persistently exceeded those in the controls throughout the course (**Figure 7B**). Additionally, our in-house scRNA-seq data also demonstrated that CD14^+^ monocytes constituted an important cellular source of CFD, with expression levels being significantly upregulated in the MG group versus the control group (**Figures 7C, D**). The result of RT-qPCR showed that CFD was upregulated in PBMC from MG patients (**Figure 7E**). Based on CFD expression status, we stratified monocytes into CFD-positive (CFD^+^) and CFD-negative (CFD^-^) groups. To elucidate the regulatory mechanisms of CFD in monocytes, we performed trajectory analysis using CytoTRACE to evaluate transcriptional heterogeneity. CFD^+^CD14^+^ monocytes showed a higher CytoTRACE score (**Figure 7F**), suggesting this subset might have a greater capacity for expansion and functional plasticity under pathological conditions. Furthermore, GSEA suggested that CFD^+^CD14^+^ monocytes were enriched in pathways related to complement and coagulation cascades, antigen processing and presentation, and proteasome pathways (**Figure 7G**). These findings hinted that CFD^+^CD14^+^ monocyte subset may contribute to MG pathogenesis by integrating innate immune with adaptive immune regulation, thereby promoting the autoreactive immune responses of MG. Compared with control group, some key genes such as DHFR, TMEM176B, CD300H, and PTSG2 were significantly elevated in CFD^+^CD14^+^ monocytes of MG group (**Figure 7H**). Upregulated differentially expressed genes were enriched in pathways related chemotaxis, the production of inflammatory cytokines (IL-1, IL-2, IL17), and IL-17 signaling (**Figures 7I, J**), which suggesting CFD^+^CD14^+^ monocytes might participate in MG through amplifying Th17-mediated inflammatory responses. Subsequently, we meticulously constructed a cell-cell communication network with CellChat. The analysis revealed that receptors on CFD^+^CD14^+^ monocytes exhibited enhanced responsiveness in both signal reception and transmission relative to CFD^-^ cells, indicating a vital role for CFD in the pathobiology of MG (**Supplementary Figure 7**). Meanwhile, CFD^+^CD14^+^ monocytes demonstrated higher interaction strength in both incoming and outgoing signaling pathways compared to CFD^-^ cells (**Figure 7K**). Comparative analysis of interaction strength in signaling networks between the MG vs control groups revealed a markedly greater difference in CFD^+^ cells than in CFD^-^ cells (**Figure 7L**). Ligand-receptor analysis identified significant signaling interactions between CFD^+^CD14^+^ monocytes and B cells, such as TNFSF13B-TNFRSF13C, SEMA4D-CD72, and LGALS9-CD44/CD45 (**Figure 7M**; **Supplementary Figure 8**). These results indicated that CFD contributes significantly to MG development by altering the biological behavior of CD14^+^ monocytes.

## Discussion

In this study, the integration of multi-omics data and machine learning has uncovered novel insights into the pathogenesis of MG, with a particular focus on the critical role of CFD^+^ CD14^+^ monocytes. Emerging evidence suggests that monocytes, beyond their classical phagocytic functions, actively shape adaptive autoimmune responses through cytokine secretion, antigen presentation, and indirect or direct cellular crosstalk (4). The abnormal proportion and transcriptional reprogramming of monocytes in autoimmune diseases imply a central role for this immune population in disease biology (29).

Accumulating evidence from scRNA-seq analysis has demonstrated the heterogeneity of monocytes and their critical role in the progression of MG. For instance, studies have reported that VISTA^+^ monocyte was an increased CD14^+^ monocyte subset in MG, with potential promoting inflammation responses (6, 30). Guo et al. identified two dysregulated monocyte subpopulations (CD14^+^S100A12^+^ and CD14^+^FOS^+^ monocytes) that exhibited dynamic changes in enrichment and inflammatory characteristics in functional cure MG patients (31), which provided promising markers over a long period of follow‐up. In addition, a mendelian randomisation study based on GWAS summary data demonstrated that upregulation of CD64, HLA‐DR and CD40 of monocyte cells contributed to MG development (32). We observed an elevated proportion of CD14^+^ monocytes among MG patients in both GSE227835 dataset and in-house scRNA-seq data. Optimal transport analysis revealed CD14^+^ monocytes displayed notably transcriptomic reprogramming in MG patients. Our findings further highlight the important role of CD14^+^ monocytes in the pathogenesis of MG.

To further explore cell type-specific risk mechanism, we integrated GWAS data and single-cell data. We identified 125 MG risk genes based on TWAS analysis, complementing existing knowledge of MG genetics and expanding the repertoire of potential pathogenic mediators. Compared to other cell types, CD14^+^ monocytes possessed higher TWAS activity. TWAS activity reflects the enrichment of genetically risk-associated gene expression, indicating that CD14^+^ monocytes might be primary targets of genetic susceptibility in MG. High TWAS activity monocytes were predominantly enriched in the MG group and exhibited elevated cytoTRACE scores, suggesting increased plasticity and functional potential. The functional enrichment of mTORC1 signaling, complement activation, and inflammatory pathways in high TWAS activity monocytes points to a metabolically and immunologically activated state. mTORC1 is a master regulator of cellular metabolism and is known to participate in the regulation of immune response (33, 34). Its co-activation with complement pathways in our data suggests a synergistic mechanism driving immune dysregulation in MG. Complement activation has been implicated in antibody-mediated pathologies, including MG (35). Our results localized complement dysregulation to a specific monocyte subset, thereby linking systemic complement activation to a cellular driver.

To better identify potential therapeutic targets for MG, we applied multiple machine learning approaches to identify feature genes and analyzed their expression levels at single cell resolution. Ultimately, CFD (complement factor D) was identified as the key gene in MG. We observed that CFD were primarily enriched in monocytes and significantly upregulated in MG group. CFD, a serine protease, is essential for the activation of the alternative complement pathway and plays a pivotal role in both its initiation and amplification loop (36). CFD catalyzes the cleavage of factor B to form the C3 convertases C3(H_2_O)Bb and C3bBb, with C3(H_2_O)Bb subsequently cleaving C3 into C3a and C3b. The alternative pathway, through a C3b- and C3-convertase–mediated amplification loop, contributes to the classical pathways (37). It has been proposed that the alternative pathway may not play a significant role in MG. However, a recent study demonstrated that higher alternative (AH50) complement pathway activity levels were correlated with earlier symptom recurrence of generalized MG (38), hinting alternative pathway may play an important role in MG. The alternative pathway contributes substantially to overall complement activation, including that mediated by the classical pathway (39). Therefore, the role of the alternative complement pathway in MG is needed to further research.

Pseudotime trajectory analysis revealed that CFD was highly expressed during the early stages, suggesting its potential involvement in the initial pathogenesis of MG. Given the potentially important role of CFD, CD14^+^ monocytes were further categorized into CFD^+^CD14^+^ monocytes and CFD^-^CD14^+^ monocytes. Compared with CFD^-^CD14^+^ monocytes, CFD^+^CD14^+^ monocytes exhibited enhanced plasticity and were enriched in complement and coagulation cascades, antigen processing and presentation, and proteasome pathways—processes critical for autoimmune pathogenesis. In addition, upregulated differentially expressed genes of CFD^+^CD14^+^ monocytes in MG were involved in pathways related chemotaxis, the production of inflammatory cytokines (IL-1, IL-2, IL17), and IL-17 signaling, further underscore their pro-inflammatory capacity, potentially contributing to the overall inflammatory milieu in MG. These finding suggested that CFD^+^CD14^+^ monocytes may promote MG through multiple synergistic mechanisms.

Cellular interaction analysis revealed that CFD^+^CD14^+^ monocytes exhibit enhanced signal reception and transmission capacity compared to CFD^-^ cells, with stronger interaction strength in both incoming and outgoing signaling pathways in MG. The enhanced cellular crosstalk of CFD^+^CD14^+^ monocytes, especially with B cells via pairs such as TNFSF13B-TNFRSF13C (BAFF–BAFF-R) and SEMA4D-CD72, provides a plausible cellular mechanism for the dysregulated B-cell responses characteristic of MG. BAFF signaling is crucial for B-cell survival and antibody production, and its blockade is therapeutic in some autoimmune diseases (40, 41). Our data suggest that CFD^+^CD14^+^ monocytes could be a key source of BAFF in MG, thereby promoting autoreactive B-cell persistence and autoantibody production. Additionally, a study demonstrated that SEMA4D-CD72 plays an important role in the activation of the proinflammatory activity of B cells (42). These findings suggest that CFD^+^CD14^+^ monocytes enhance B cell-mediated autoimmunity through direct intercellular communication, reinforcing the critical crosstalk between innate and adaptive immunity in MG. Given the well-established role of B cells and autoantibodies in MG pathogenesis, the enhanced interaction between CFD^+^CD14^+^ monocytes and B cells provides a novel mechanism by which innate immune dysregulation drives adaptive immune responses in MG.

This study has some limitations. First, while the integration of multi-omics data and machine learning provides robust insights, functional validation of the identified signature genes (particularly CFD) *in vitro* and *in vivo* is needed to confirm their pathogenic role. CFD^+^CD14^+^ monocytes are a model driven candidate subset that requires functional validation in future studies. Second, the study focused on peripheral blood monocytes, and future studies should investigate CD14^+^ monocytes in tissues relevant to MG (e.g., thymus and neuromuscular junction) to determine their local pathogenic contributions, which may reveal additional tissue-specific risk genes. Despite these limitations, our findings have important implications for MG therapy. The expression levels of TWAS genes observed in scRNA-seq may also reflect disease-state or environmental influences, not only genetic regulation. Targeting CFD or CFD^+^ CD14^+^ monocytes might inhibit complement activation, reduce inflammatory signaling, and disrupt pathogenic intercellular communication, addressing multiple key pathways in MG pathogenesis.

In summary, this study systematically delineates the important role of CFD^+^ CD14^+^monocytes in MG pathogenesis through integrative multi-omics and machine learning analyses. These findings provide novel insights into MG immunopathology and identify potential therapeutic targets, paving the way for the development of more effective and targeted treatments for MG.

## Data Availability

The datasets presented in this study can be found in online repositories. The names of the repository/repositories and accession number(s) can be found in the article/**Supplementary Material**.
