# Supplementary material for: CFD+CD14+ monocytes: potential pathogenic subset in myasthenia gravis uncovered by multi-omics integration and machine learning analysis
Source: Front Immunol. 2026 May 1;17:1813107. doi: 10.3389/fimmu.2026.1813107 (PMC13176296; doi:10.3389/fimmu.2026.1813107)
Supplement: Supplementary file 1 [file DataSheet1.docx]

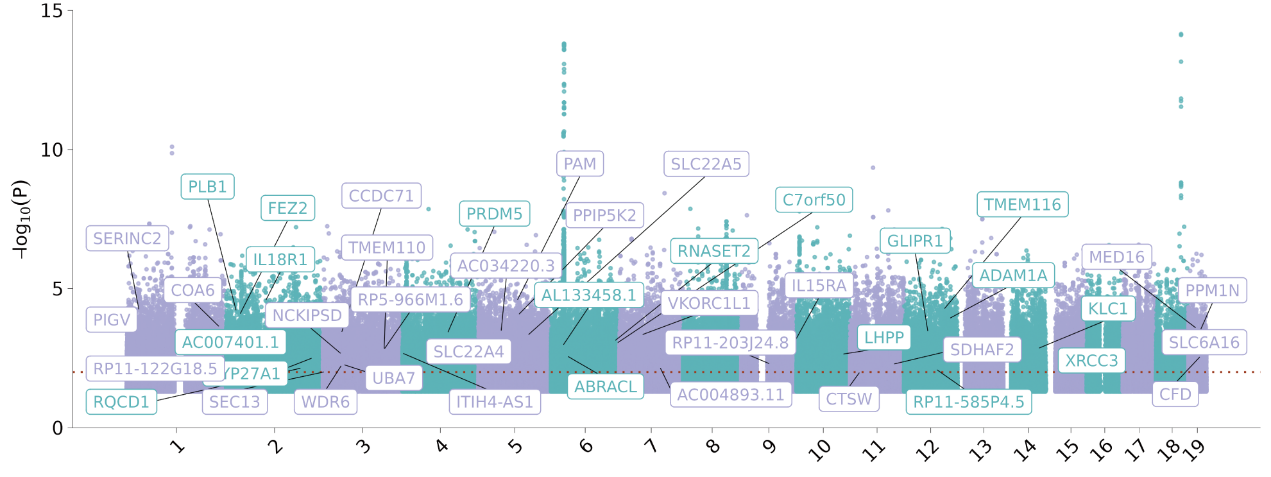


Figure S1. Rect Manhattan plot of TWAS risk genes for MG (P < 0.05).


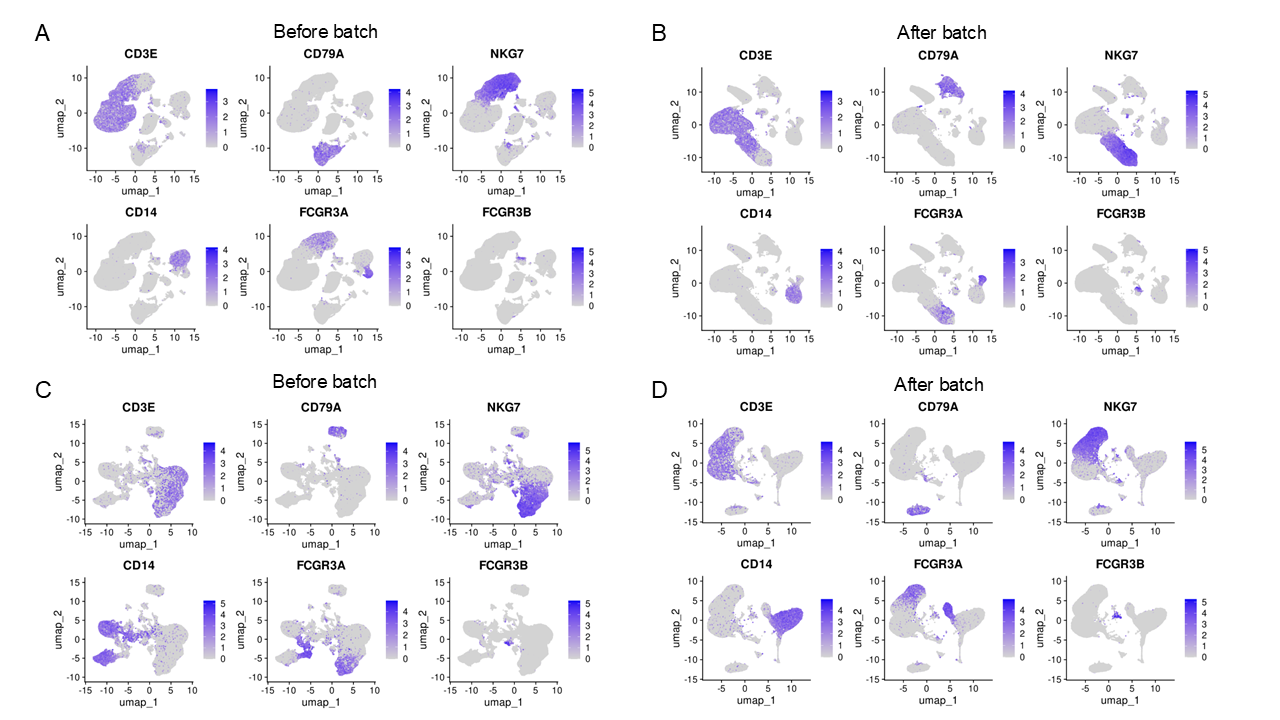


Figure S2. (A) Gene makers of major cell-type before moving batch in GSE227835 data. (B) Gene makers of major cell-type after moving batch in GSE227835 data. (C) Gene makers of major cell-type before moving batch in in-house scRNA data. (D) Gene makers of major cell-type after moving batch in in-house scRNA data.


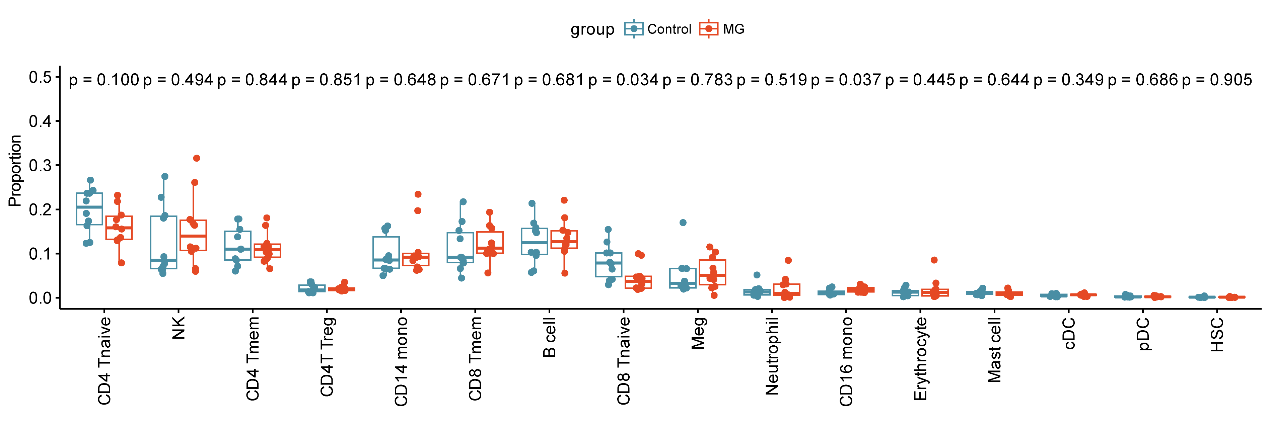


Figure S3. The proportion difference of each cell type between MG vs control (used GSE227835 dataset).


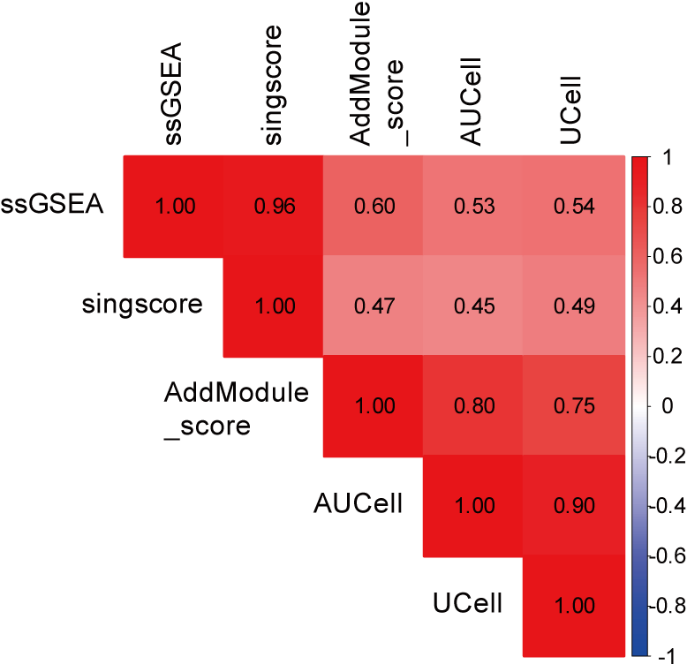


Figure S4. Heatmap showing pairwise correlations of scores from the five algorithms.


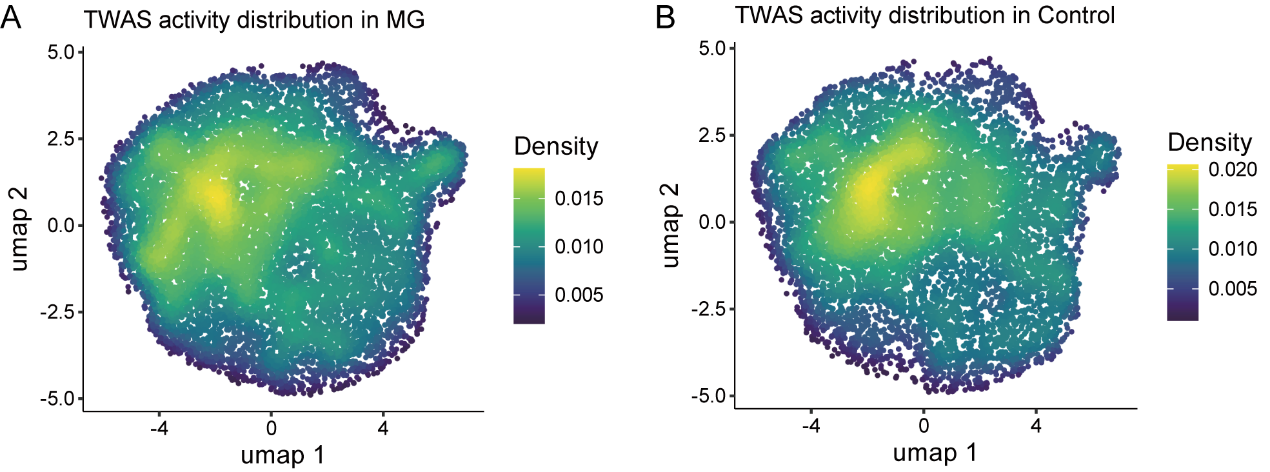


Figure S5. (A) The density distribution of TWAS activity in CD14^+^ monocytes of MG group. (B) The density distribution of TWAS activity in CD14^+^ monocytes of control group.


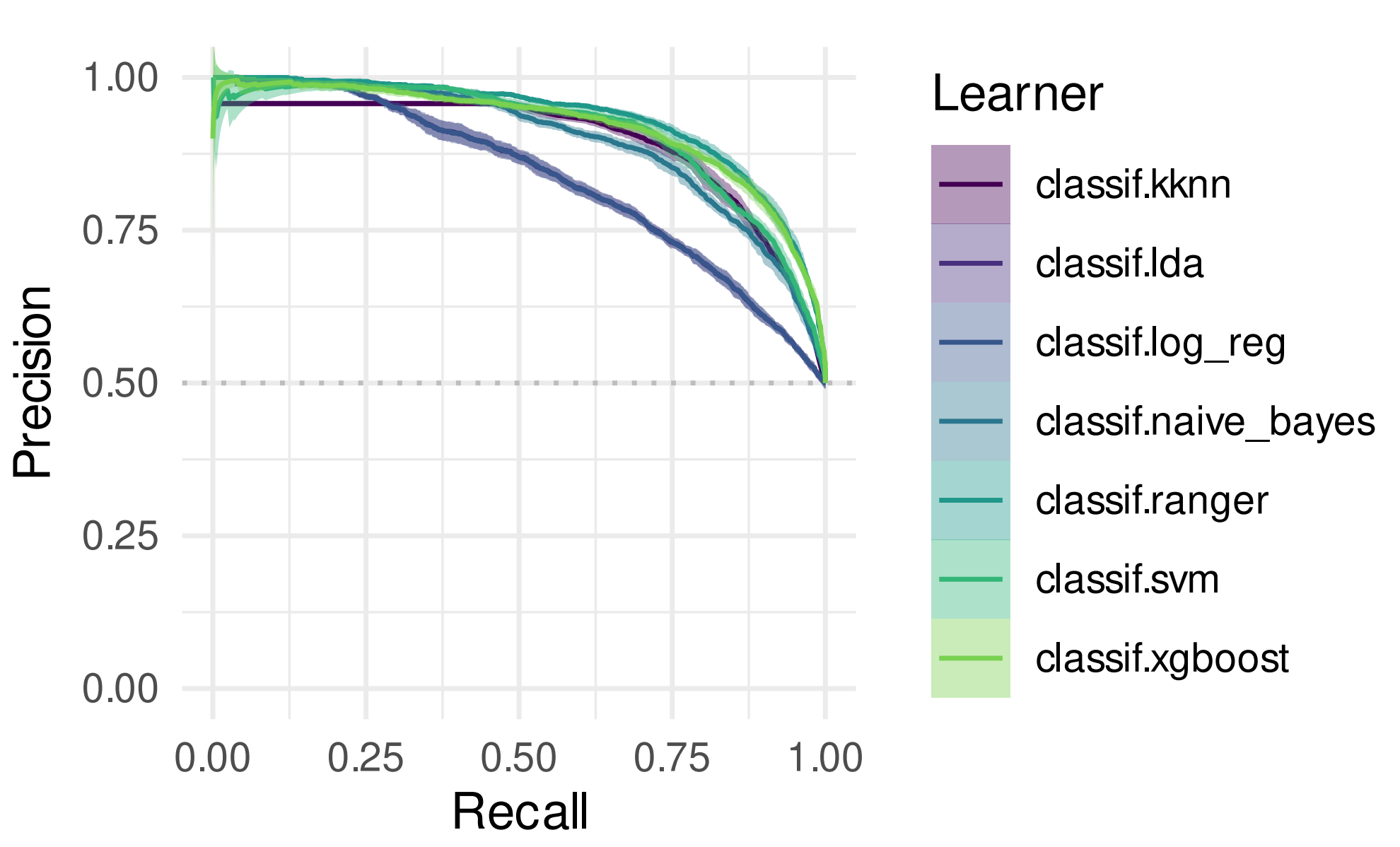


Figure S6. Precision-Recall curves for machine learning model benchmark.


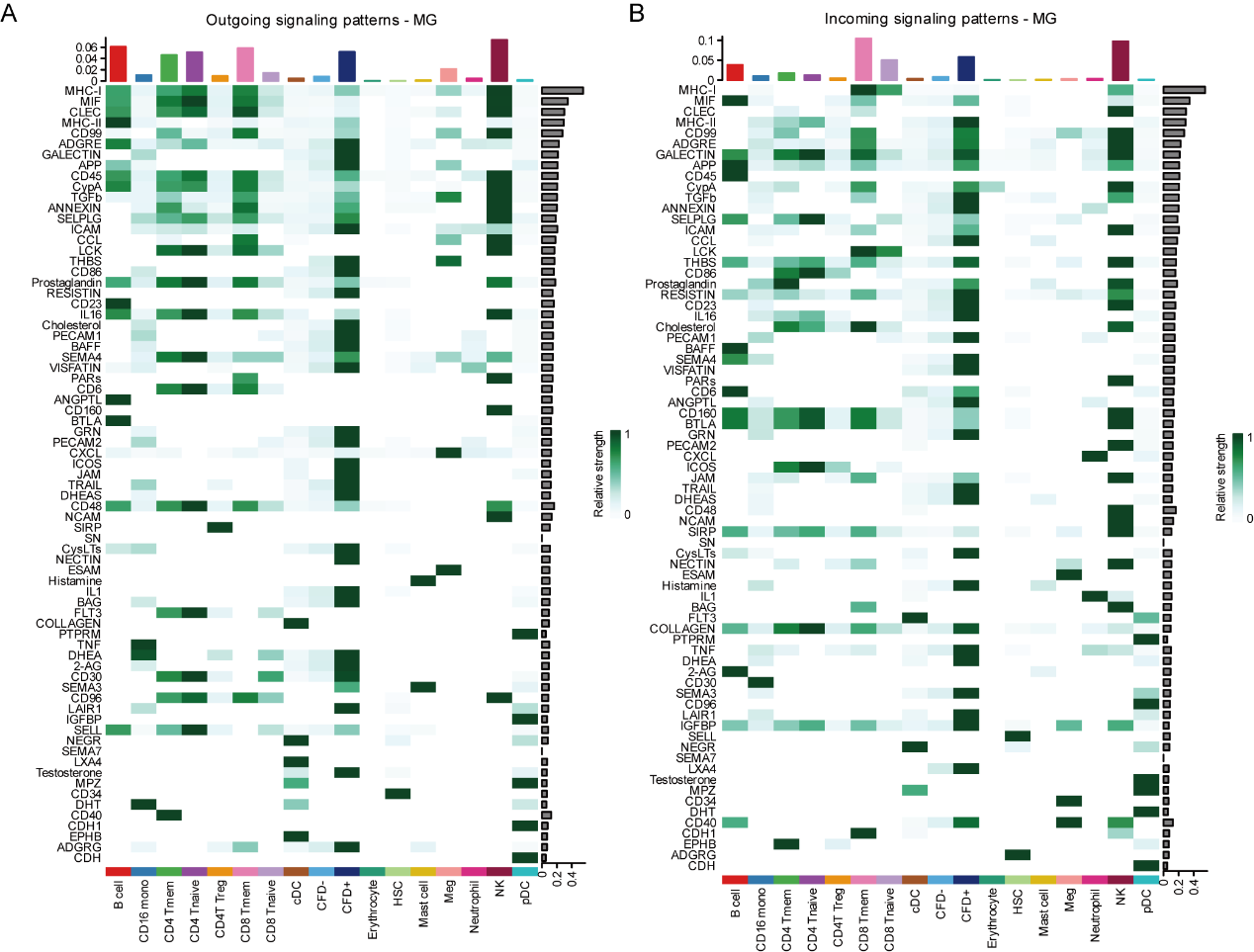


**Figure S7. Signaling role analysis on the cell–cell communication network from all signaling pathways between all cell types.**

**
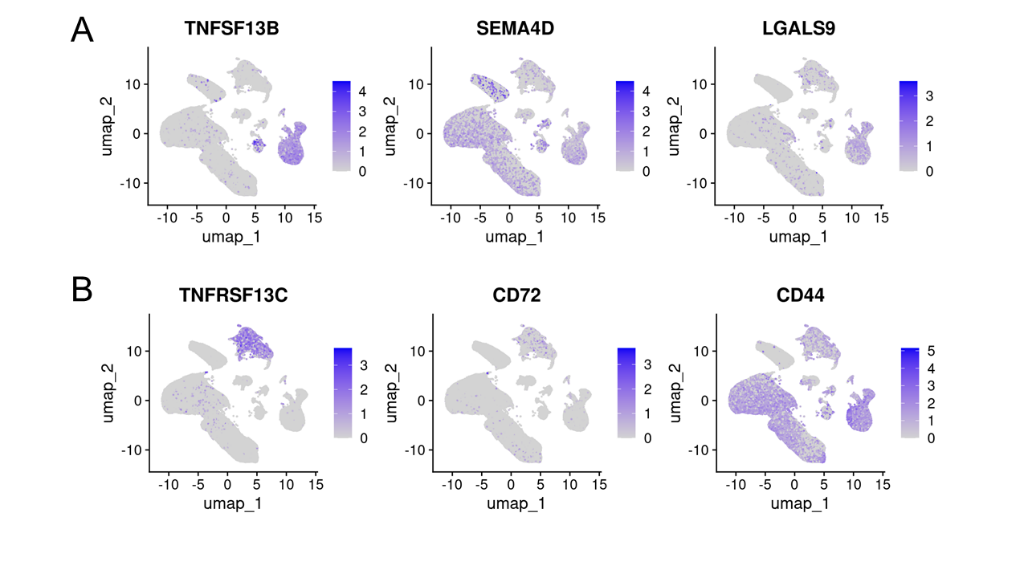
**

**Figure S8. UMAP plots showing the expression of Ligand-Receptor pairs.**
